# Consequences of Landscape Fragmentation on Lyme Disease Risk: A Cellular Automata Approach

**DOI:** 10.1371/journal.pone.0039612

**Published:** 2012-06-25

**Authors:** Sen Li, Nienke Hartemink, Niko Speybroeck, Sophie O. Vanwambeke

**Affiliations:** 1 Georges Lemaître Centre for Earth and Climate Research, Earth and Life Institute, Université catholique de Louvain, Louvain-la-Neuve, Belgium; 2 Institute of Health and Society (IRSS), Université catholique de Louvain, Brussels, Belgium; Kansas State University, United States of America

## Abstract

The abundance of infected Ixodid ticks is an important component of human risk of Lyme disease, and various empirical studies have shown that this is associated, at least in part, to landscape fragmentation. In this study, we aimed at exploring how varying woodland fragmentation patterns affect the risk of Lyme disease, through infected tick abundance. A cellular automata model was developed, incorporating a heterogeneous landscape with three interactive components: an age-structured tick population, a classical disease transmission function, and hosts. A set of simplifying assumptions were adopted with respect to the study objective and field data limitations. In the model, the landscape influences both tick survival and host movement. The validation of the model was performed with an empirical study. Scenarios of various landscape configurations (focusing on woodland fragmentation) were simulated and compared. Lyme disease risk indices (density and infection prevalence of nymphs) differed considerably between scenarios: (i) the risk could be higher in highly fragmented woodlands, which is supported by a number of recently published empirical studies, and (ii) grassland could reduce the risk in adjacent woodland, which suggests landscape fragmentation studies of zoonotic diseases should not focus on the patch-level woodland patterns only, but also on landscape-level adjacent land cover patterns. Further analysis of the simulation results indicated strong correlations between Lyme disease risk indices and the density, shape and aggregation level of woodland patches. These findings highlight the strong effect of the spatial patterns of local host population and movement on the spatial dynamics of Lyme disease risks, which can be shaped by woodland fragmentation. In conclusion, using a cellular automata approach is beneficial for modelling complex zoonotic transmission systems as it can be combined with either real world landscapes for exploring direct spatial effects or artificial representations for outlining possible empirical investigations.

## Introduction

Risk of vector-borne diseases is highly dependent on the abundance of infected vectors [1], [2]. Lyme borreliosis, the most frequent vector-borne disease of humans in temperate zones [3], is no exception. The causative agent is the spirochaete *Borrelia burgdorferi* sensu lato, which is transmitted by *Ixodes ricinus* and *I. persulcatus* in Europe and *I. scapularis* in North America. Each post-egg developmental stage of ticks (larva, nymph and adult female) takes one blood meal lasting several consecutive days, followed by potentially prolonged interstadial development. *B. burgdorferi* is transmitted between ticks and vertebrate hosts over the course of the blood meal. A broad range of transmission-competent vertebrate hosts (so-called reservoir hosts), such as rodents, insectivores and several bird species [4], [5], along with ticks, contribute to the maintenance of transmission. Medium-sized mammals, such as hares, and large mammals, like game, cattle and horses, are reservoir-incompetent but nevertheless important as they facilitate survival by providing blood meals to large numbers of adult ticks and thereby contribute to higher tick abundance [6]. Preferential habitats of ticks and many of their hosts occupy a large fraction of rural landscapes and more suitable conditions can be found in certain land cover types, or in certain arrangements of landscape elements.

Landscape fragmentation can be important in shaping disease patterns. First, landscape fragmentation may lead to an uneven spatial distribution of pathogen, by subdividing hosts into subpopulations of varying size, an important element of disease transmission. Subpopulations varying in size may have different patterns of pathogen persistence. Second, when pathogens are established in a subpopulation, the spread of the pathogen across the landscape may be slowed or prevented by fragmentation as it interferes with host movement. This is particularly relevant for tick-borne diseases, which can be transported over long distances only by moving hosts.

The effects of landscape fragmentation on the dynamics of vector-borne zoonoses have been receiving increasing attention in the last decade. Associations between landscape structure, tick abundance and disease incidence have been found in various contexts. The extent of habitats, as well as their relative positions and transition areas (i.e. ecotones) contributed to high levels of tick-borne encephalitis incidence in the Latvian countryside [7] and of *I. ricinus* abundance in northern Spain [8]. *I. ricinus* abundance was found positively related to the length of forest edge in northern Belgium [9] and significantly associated to isolation and permeability of woodland patches in central Spain [10]. *B. burgdorferi* sensu lato was found more prevalent in *I. ricinus* populations in fragmented woodland with low patch surface area and high edge density in central France [11] and in well-connected vegetation patches in the western Palearctic region [12]. Similar observations were made with regard to *I. scapularis* [13], [14], [15], [16]. Determining the key factors underpinning the spatial variation in disease risks would facilitate the development and management of preventive measures. In this study, we aimed at (i) investigating the effects of woodland fragmentation on infected tick abundance and (ii) identifying key landscape characteristics associated with risks of Lyme disease. Our secondary objective was to develop a spatially-explicit model for understanding the spatial dynamics of tick-borne diseases. A set of artificial woodland patterns were tested with two different types of adjacent land cover (i.e. non-vegetated area and grassland). These patterns were later examined for a set of landscape characteristics and their associations to the density and infection of ticks.

## Materials and Methods

### Model and Parameters

The model was developed using a cellular automata approach. Space in the model was two-dimensional, rectilinear, and organized by cells. A time step of one week and a cell size of one ha were adopted and we focused on a landscape dimension of 50×50 cells. Populations of ticks and hosts, and habitat type constituted three layers of cell attributes:

- Tick population layer. The three post-egg tick life stages were considered: larva, nymph, and adult. Total and infectious populations in both questing and feeding phases were stated for each cell.
- Host population layer. Total populations of two generalised types of hosts were stated, reservoir hosts and reproduction hosts. As an important component of disease transmission, infectious reservoir host population was also stated. Larvae only fed on reservoir hosts, adults only fed on reproduction hosts, while nymphs could feed on both host types. The numbers of host were stable overall but were allowed to vary in space and time within the space modelled in relation to movements. Thus, host distributions vary between time steps.
- Landscape layer. A simple “woodland” – “grassland” – “non-vegetated” structure was applied with each cell covered with one of the three classes. Host movement patterns differed between land cover types. Both woodland and grassland were assumed to be suitable habitats for ticks and reservoir hosts. However, grasslands are less suitable for ticks. Reproduction hosts may enter grassland for short stays (i.e. a proportion of a time step) but then return to their woodland habitat. Non-vegetated area relates to harvested or damaged woodland or grassland (for example by fire). Both types of host could venture into but not stay in non-vegetated area. Thus, they return in the same time step and no ticks could drop off in non-vegetated area. The following model parameters varied with land cover types: survival rates of ticks, densities of hosts and movement patterns of hosts.

Transition rules concerning tick development, pathogen transmission and host movement patterns were applied sequentially to every cell, during each successive discrete time step. In each time step, cells in the grid were examined in order (i.e. from west to east and from south to north), and their states were updated simultaneously after the transition rules had been applied to the current configuration.

#### Tick population dynamics

To achieve an accurate representation of the spatio-temporal patterns of ticks and infection patterns, tick developmental stages must be modelled in considerable detail [17]. Our model for the tick population dynamics is very similar to the approaches used by Ogden et al. [18] for *I. scapularis*, and Hoch et al. [19] and Hancock et al. [17] for *I. ricinus*. However, contrary to these models, our model is spatially explicit and takes into account the spatial and temporal population dynamics of ticks and hosts in each cell. While the model structure is generic, parameter values were based on the literature pertaining to *I. ricinus.* The population dynamics discussed in this section are assumed to be the same for infectious and non-infectious ticks. The method for including transmission dynamics is explained in detail in the section on pathogen transmission.

**Figure 1:**
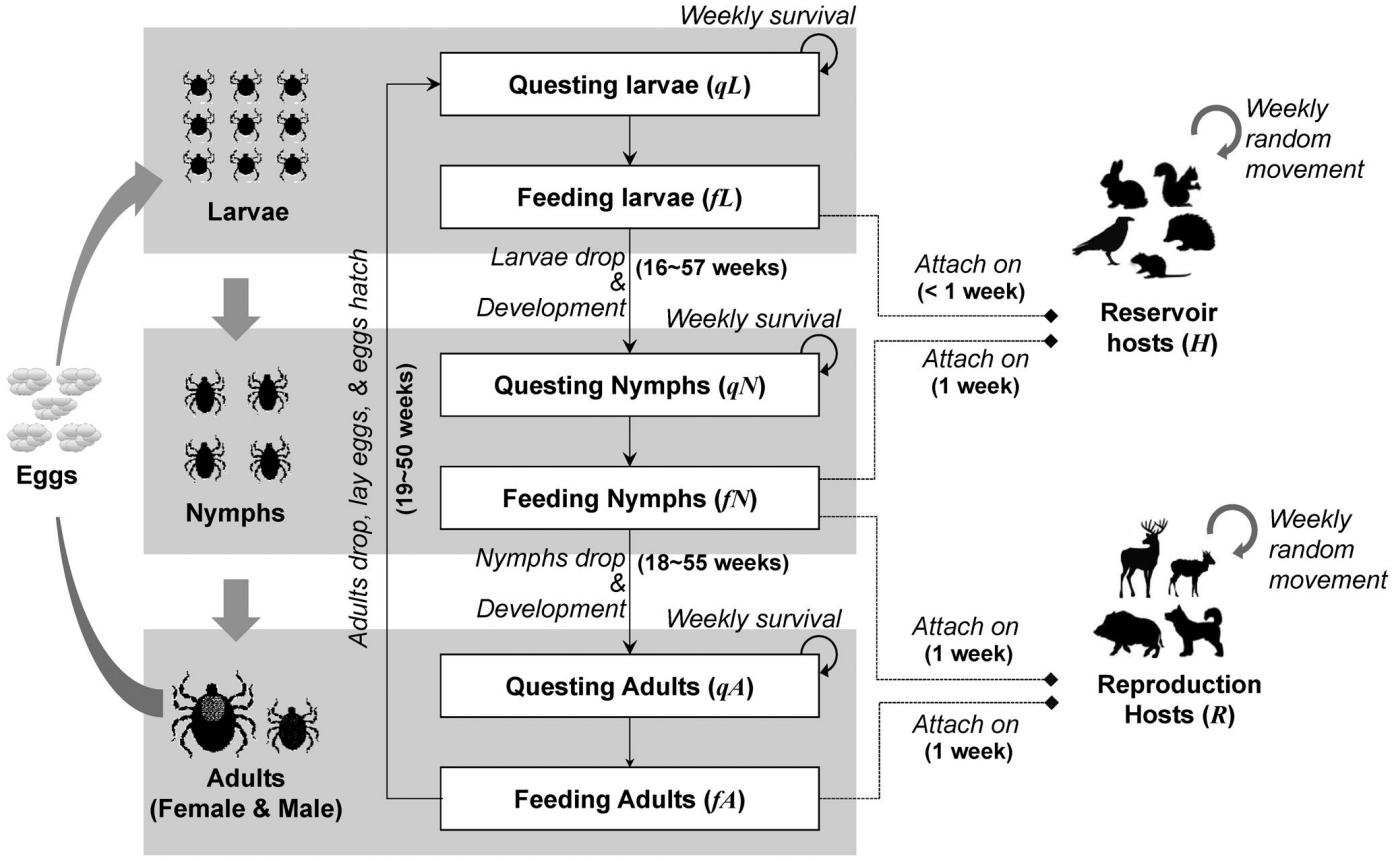
Tick life stages and their relations to host types. Solid boxes indicate populations stated in the CA model. Solid arrows indicate the development of tick populations. Dashed lines show attachment relations. Two phases, questing and feeding, were stated for each post egg life stage. Host preferences of questing ticks differ between life stages. In the model, it was assumed that larvae feed on small-sized animals, adults feed on large-sized animals, and nymphs feed on both.

Figure 1 summarizes the development of ticks and the role of both host types. In the cellular space, there are two major types of individuals for which we model the population in each cell: the ticks and the hosts. The ticks are subdivided in larvae (*L*), nymphs (*N*) and adults (*A*); and the hosts are subdivided in reservoir hosts (*H*) and reproduction hosts (*R*). Ticks can be either in questing (*q*) or in feeding (*f*) phase.

For each cell, the change in the questing tick population of a life stage at a time step was computed by adding the ticks that emerged (moulted in the previous time step from a previous life stage), and by subtracting ticks that died in the previous time step and ticks that successfully attached to hosts during the current time step (formulae in Appendix S1). Three key sets of parameters were used: (i) survival rates, (ii) feeding rates, and (iii) durations of interstadial development phases (Table 1). In this model, two categories of constant tick survival rates in woodland were used respectively for ticks in questing phases (*s^qL^, s^qN^, s^qA^*) and in the periods between feeding and moulting into the next life stages (*s^LN^, s^NA^, s^AL^*). Since tick survival rates can be significantly influenced by abiotic factors such as vapour pressure deficit, survival rates differed between the various land cover types [20], [21]. Following Hoch et al. [19], a scaling factor *F_GL_* was used to differentiate survival rates in the various land covers considered. As estimated from Mount et al. [22], survival rates of questing and developing ticks were 6% lower in grassland than in woodland, i.e. *F_GL_ = *0.94. The number of feeding ticks was estimated by *fT*  =  *a^T^·X*, where *T* refers to a tick life stage (*L*, *N*, *A*), *X* refers to the preferable host type of the tick life stage (*H*, *R*), and *a* is the average number of ticks attached to a host, which was also constant. In the case of nymphs, both host types were considered in the estimation. If the estimation was higher than the total number of questing ticks, all questing ticks were assumed to be picked up. Larvae were assumed to need less than one week to complete a blood meal (around three days according to Macleod [23]), and nymphs and adults to need one week [17]. Finally, durations of interstadial development period (*d^LN^*, *d^NA^*, *d^AL^*) used in the model were set constant for the present study.

**Table 1:** Parameters used in the model.

Symbol		Definition	Value	Range	Source
*Parameters in relation to tick life cycle*					
*a^L^*	Average no. of larvae on one reservoir host		8	0 ∼ 30	[66], [67]
*a^NH^*	Average no. of nymphs on one reservoir host		0.6	0 ∼ 2	[66], [68]
*a^NR^*	Average no. of nymphs on one reproduction host		0.95	0 ∼ 32	[69], [70]
*a^A^*	Average no. of adults on one reproduction host		6	0 ∼ 28	[69], [71]
*s^qL^*	Weekly survival rate of questing larvae in woodland		0.96	0.95 ∼ 0.99	[72]
*s^qN^*	Weekly survival rate of questing nymphs in woodland		0.99	N/A	[72]
*s^qA^*	Weekly survival rate of questing adults in woodland		0.99	N/A	[72]
*β*	Average no. of eggs per adult		2000	1500 ∼ 2500	[73]
*s^LN^*	Survival rate from feeding larvae to questing nymphs in woodland		0.8	0 ∼ 0.89	[17], [72], [74]
*s^NA^*	Survival rate from feeding nymphs to questing adults in woodland		0.8	0.0 ∼ 0.93	[17], [72], [74]
*s^AL^*	Survival rate from feeding adults to questing larvae in woodland		0.45*	0.0 ∼ 0.49	[17], [72], [74]
*F_GL_*	Scaling factors for questing and developing ticks survival rates in grassland		0.93	N/A	[22]
*d^LN^*	Duration of development period from feeding larvae into questing nymphs (week)		46	16∼ 57	[72], [74]
*d^NA^*	Duration of development period from feeding nymphs into questing adults (week)		54	18∼ 55	[72], [74]
*d^AL^*	Duration of development period from feeding adults to questing larvae (week)		46	19∼ 50	[72], [74]
*Parameters in relation to pathogen transmission*					
*θ^HT^*		Transmission efficiency from reservoir host to larva and nymph	0.6	0.1 ∼ 0.9	[73]
*θ^TH^*		Transmission efficiency from larva and nymph to reservoir host	0.9	0.8 ∼ 1	[75]
*θ^TE^*		Transmission efficiency from adult to egg	0.01	0 ∼ 0.01	[76]
*r^H^*		Weekly removal rate of infection in reservoir host population due to mortality	0.04	0.03 ∼ 0.12	[77]
*Parameters in relation to host movement patterns*					
*MC^H^*		Movement capacity of reservoir host per week (m)	100	0 ∼ 150	[29]
*MC^R^*		Movement capacity of reproduction host per week (m)	500	0 ∼ 4000	[30]
*pG*		Proportion of time step spend in grassland for reproduction host (%)	35	9 ∼ 77	[33]
*Parameters in relation to landscape heterogeneity*					
*d^H^*		Density of reservoir host in woodland and grassland (ha^−1^)	75	0 ∼ 135	[34]
*d^R^*		Density of reproduction host in woodland (ha^−1^)	0.15	0 ∼ 34	[35], [36]

N/A =  Not Applicable.

* Assuming 50% of adults are females produce hatched larvae.

#### Pathogen transmission

Generally, tick-borne pathogens can be transmitted by three different routes: (i) systemic, the pathogen is transmitted during the blood meal taken by an uninfected tick on an infected host or by an infected tick on an uninfected host; (ii) non-systemic, the pathogen is transmitted between co-feeding ticks [24], (iii) transovarial, ticks hatch from infected eggs [25]. Since the significance of non-systemic transmission on the maintenance of *Borrelia spp.* remains unclear [26], [27], only systemic and trans-ovarial transmissions were considered in this study.

Assuming that adults do not feed on reservoir hosts, systemic transmission only takes place between reservoir hosts and feeding ticks in larval and nymphal stages (formulae in Appendix S1, parameters in Table 1). For each cell in time step *t*, we modelled the increased infectious subset of each population concerned (*fIT*: feeding infectious larvae and nymphs, *IH*: infectious reservoir hosts) based on classical SI models: Δ*fIT*_t_  =  *θ^HT^·*(*IH_t_*/*H_t_*)*·*(*fT*_t_-*fIT_t_*); Δ*fIH*_t_  =  *θ^TH^· fIT*_t_ *·*(*H_t_-IH_t_*)/*H_t_,* where *θ^HT^* and *θ^TH^* are constant and denote the pathogen transmission efficiencies (i.e. the proportion transmitted) from infectious reservoir hosts to susceptible ticks and from infectious ticks to susceptible reservoir hosts. As ticks only feed once per life stage, ticks getting infected through systemic transmission could only become infectious in the next life stage. Besides, the population of questing infectious larvae can be increased only via trans-ovarial transmission. A constant parameter *θ^TE^* referring to the pathogen transmission efficiency from an infectious adult female to her eggs was used for this purpose. Finally, an average weekly removal rate (*r^H^*) of infectious reservoir hosts was considered based on the life span of reservoir host [28].

#### Host movement patterns

Two modes of host movement were considered. The first mode concerned the movement in habitats, i.e. reproduction hosts moving in woodland and reservoir hosts moving in both grassland and woodland. This type of movement is modelled using parameters for the movement capacities of reservoir hosts (*MC^H^*) and reproduction hosts (*MC^R^*) in their habitat. These parameters reflect the maximum projected distance on the 2D axis per time step and were set to 100 m (1 cell) and 500 m (5 cells) respectively (Table 1). These values are based on field observations: the bank vole *Myodes (Clethrionomys) glareolus* [29], a reservoir host has a home range in the order of magnitude of 1 ha, while the roe deer *Capreolus capreolus* [30], a reproduction host, has a home range of around 100 ha. A random host movement was considered enabling the hosts to re-distribute every time step into habitats within their home range. The second mode of host movement concerned the movements between habitat and non-habitat, i.e. reproduction hosts moving between woodland and grassland. Reproduction hosts such as roe deer *Capreolus capreolus* venture into open grasslands to forage [31]. However, as human disturbance is higher in such land covers [32], they eventually return to woodland. We assumed hosts only spend a proportion of the time step in grassland (*pG*) [33]. A detailed illustration of these methods is presented in Appendix S2.

Ticks can be transported by moving hosts. Nymphs and adults, taking one week to complete their blood meals, could be transported between cells. As larvae were assumed to complete their blood meal in less than one week, the transport of larva was considered to be negligible. Along with the displacement of a proportion of hosts between habitats, the same proportion of total and infectious feeding ticks was transported. Similarly, when reproduction hosts had spent a proportion of a time step in grassland, a proportion of nymphs and adults feeding on them dropped off, and a proportion of questing nymphs and adults were picked up. Questing larvae can thus increase in both grassland and woodland after engorged adults brought in by reproduction hosts drop and reproduce.

#### Landscape heterogeneity

As the rodent density ranges from 0 to 135 rodents/ha in southern Belgium [34], a medium level of 75 per ha was assumed for reservoir hosts in both woodland and grassland. A population density of reproduction hosts of 0.15 per ha in woodland was estimated by dividing the total number of game animals in Wallonia (approximately 750000 heads of red deer, roe deer, fallow deer, mouflons and wild boars in 2008 [35]) by total woodland area in Wallonia (5000 km^2^ [36]). In Europe, the densities of nymphs in woodland can be over 10000 nymphs/ha [3] and the infection prevalence of *B. burgdorferi* s.l. in nymphal population has been found greater than 20% [37]. These values were adopted to initialise the model.

### Model Implementation

The model was built using Repast Simphony, a cellular automata and agent-based modelling toolkit based on JAVA. A torus boundary was considered for the cellular space. In each time step, tick populations were updated first. Development and survival of tick infections and populations were calculated for each cell at each life stage (Figure 1). After that, tick attachment was examined, and systemic pathogen transmission took place between ticks and reservoir hosts. Finally, host movements were considered and feeding nymphs and adults were transported. For host movement in habitat (i.e. reservoir host moving in grassland and woodland, and reproduction host moving in woodland), attached ticks on the out-moving animals were all dropped off at the end of the step.

### Model Validation

**Figure 2:**
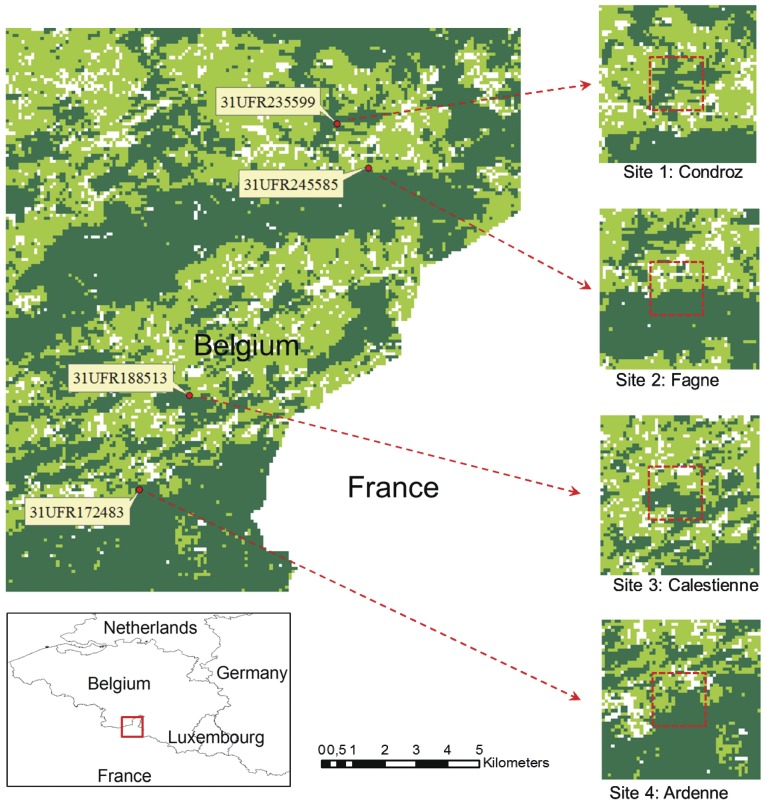
Land cover map of the study areas of Misonne et al. [**38**] **.** Light green: grassland; dark green: woodland; white: no or sparse vegetation. Study sites are tagged with coordinates (military grid reference system) and pointed to the corresponding site maps and site names. The size of cell is 1 ha. Highlighted zones in site maps refer to the sample blocks.

A set of simulated results was compared with the study by Misonne et al. [38], in which nymphal and adult *I. ricinus* ticks were collected from four forested sites in southern Belgium in July 1996, and examined by PCR for *B. burgdorferi* s. l. For the purpose of validation, a land cover map (Figure 2, cell size 1 ha) with three land cover types (woodland, grassland and non-vegetated area) of all study areas was used in the cellular automata model. Woodland included broadleaf and coniferous forests. Grassland included pasture and moorland. Non-vegetated area included peri-urban and water area, or harvested vegetation and deforested area. The four study sites were located based on the coordinates (exact to 100 m) provided in [38]. To assess the model performance at different spatial scales, Lyme disease risk indices (i.e. density of nymphs (DON), nymphal infection prevalence (NIP), and their product, density of infectious nymphs (DIN)) were calculated at two levels for each site: (i) site map level, a site map (50×50 cells) was extracted, and (ii) sample block level, a 10×10 cells map centred on the coordinates.

### Sensitivity Analysis

The sensitivity of the model to all parameters (Table 1) was assessed using the risk indices (DON, NIP and DIN) as outcomes. All simulations were performed with site map 1 (site name: Condroz, Figure 2). For each parameter (*P*), the following sensitivity index was calculated [18], [39]: *S  =  Log*_10_(*LRI_i_*/*LRI*_0_)/*Log*_10_(*P_i_*/*P*_0_), where *LRI_0_* is a Lyme disease risk index at equilibrium when using the default values for all parameters (Table 1), and *LRI_i_* is the same Lyme disease risk index when the parameter value is increased from its default value *P_0_* to *P_i_*. Variations of +5% were applied to parameters, except for: (i) weekly survival rates of tick life stage, for which variations of (+5%) were tested for mortality, as a survival rate greater than 1 is meaningless, and; (ii) durations of development which were modified by +1 week. It should be noted that, the increases of 5% in movement capacities (5 m and 25 m for reservoir and reproduction hosts) only increased the probabilities of out-moving populations but did not increase the home ranges, for which a minimum increase of 100 m is needed. Higher *S* indicates a stronger effect of the change on the parameter on the risk indices. Values of 1 or −1 indicate a linear effect.

### Effect of Woodland Fragmentation on Lyme Disease Risk Indices

#### Fragmentation scenarios

**Figure 3:**
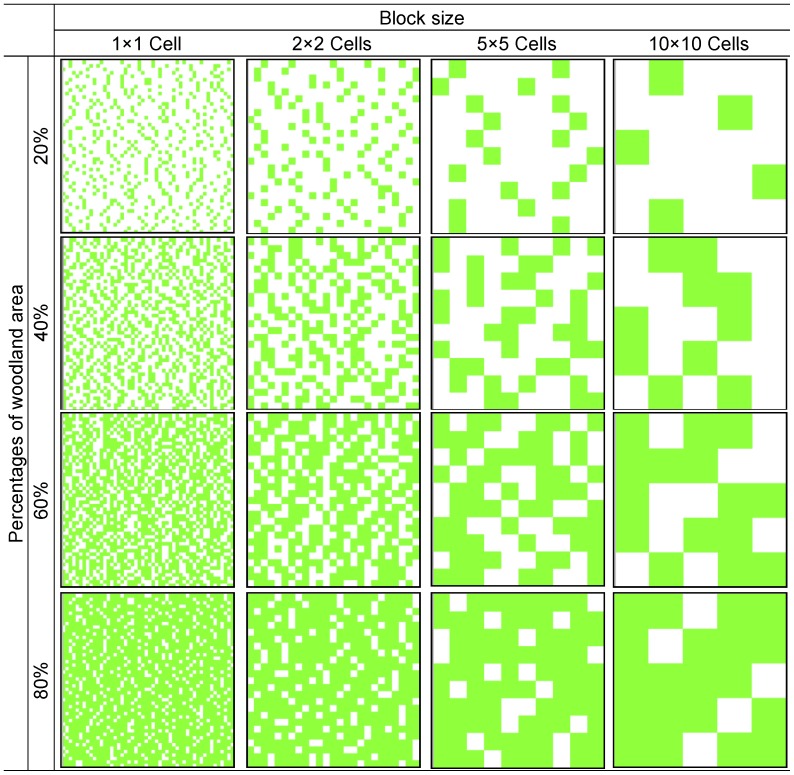
Examples of landscapes fragmented in different scenarios. Green cells refer to woodland areas. White cells refer to non-vegetated areas in situation I and grassland areas in situation II. The dimension of each landscape is 50×50 cells and the size of cell is 1 ha.

Lyme disease risk indices of DON, NIP and DIN in woodland were set as the outputs of the model. With regards to the surroundings of woodland, two situations (I & II) were hypothesised: non-vegetated area and grassland. For each situation, we applied different scenarios with respect to the percentage covered with woodland (20%, 40%, 60 and 80%) and with respect to the size of block (i.e. the basic unit square). The distribution of the blocks of woodland is randomly generated for each simulation. The block sizes applied were 1×1 cell (which is well within movement capacities of both hosts types), 2×2 cells, (meaning that reproduction hosts can reach the nearest neighbouring woodland patches, as the smallest between patch distance is one block, while reservoir hosts cannot), 5×5 cells and 10×10 cells, in which neither types of host can reach the nearest neighbouring woodland patches. In total, 16 scenarios of woodland fragmentations were thus considered. Scenarios were applied to an artificial landscape of 50×50 cells (Figure 3). Five landscapes were randomly generated for each scenario to assess the variability of outcomes. 80 landscapes were thus generated for each situation, for a total of 160 landscapes. Finally, 160 simulations were performed to record the risk indices, thus one for each landscape.

#### Statistical analysis of model outcomes

Beyond percentage cover and patch size, other aspects of landscape structure varied between scenarios, e.g. patch shape. In the final step, associations between DON, NIP and DIN and landscape configuration metrics were tested. First, the configuration of the 160 landscapes was characterised using Fragstats™ software. Three metrics were obtained at landscape level for woodland: (i) patch density, the number of patches per surface unit; (ii) shape index, which relates patch perimeter to a standard shape and increases with increasing shape complexity; and (iii) aggregation index, which relates the observed number of like adjacencies to the maximum possible number of like adjacencies. DON, NIP and DIN were regressed against patch density, shape index and aggregation index using linear, power and exponential regressions.

## Results

Outcomes were recorded after 1560 simulated weeks (30 years) for validation, sensitivity analysis and simulation.

### Comparison with Misonne et al. [38]

The simulated tick density and infection prevalence were in general in good agreement with the observations of Misonne et al. [38]. Density and infection prevalence of nymphal and adult ticks in all site maps (50×50 cells) as well as in sample blocks (centred, 10×10 cells) achieved equilibrium values for at least five simulated years (changes between time steps were smaller than 0.5%). For all site maps, average densities of 7100±20 adults and 65900±100 nymphs per ha were obtained, which falls within the estimated actual density range from 67000 to 120000 nymphs and adults per ha. This range was calculated by assuming a 5%∼9% sampling efficiency [40] for the dragged density provided in Misonne et al. [38] (i.e. 6000 nymphs and adults per ha). The simulated ratio of adults to nymphs was 0.11 which is close to but slightly higher than 0.10 from the field. Tick abundances were not compared for each site as information on the sizes of sampling areas and duration of sampling periods were missing from the article. The average infection prevalence of nymphs over the four site maps was 22.5%±1.7% (simulated) vs. 22.3% (99 infectious out of 444 trapped), and that of adults was 43.1%±2.8% (simulated) v.s. 33.3% (15 infectious out of 45 trapped). At the level of sample blocks, simulated nymphal infection prevalence failed to reflect the differences among sites. The averaged values in the last five simulated years were respectively 22.6%±0.5%, 25.1%±0.2%, 20.5%±0.1% and 22.3%±0.5% compared to 19%, 28%, 22% and 20% from the field. (Test of equal proportions: X^2^ = 0.63, p-value  = 0.8). No other field information was available for further investigation. Such difference among sites could be due to the composition and abundance of local host community (c.f. the results of sensitivity analysis). Adult infection prevalence was not considered at that level as sample sizes were small (8, 12, 23 and 2 adults trapped in each site). These results indicate a good performance of the model only at site map level.

### Sensitivity Analysis

**Figure 4:**
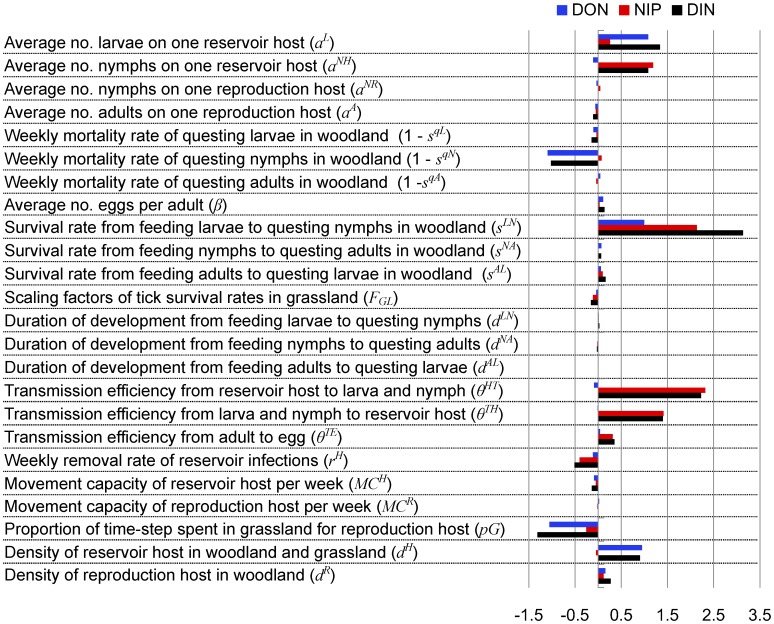
Model sensitivity results. Bar chart indicates the value of sensitivity index (*S*) on Lyme disease risk indices examined for each parameter. Black, red and blue bars refer to *S* values on density of nymphs (DON), nymphal infection prevalence (NIP), and density of infectious nymphs (DIN) respectively.

The stochastic approach for host movements leads to uncertainty in the sensitivity analysis. The pattern of sensitivity was consistent when testing parameters with different size of variations, suggesting *S* was a robust indicator of sensitivity, similarly to findings by Keeling and Gilligan [39]. The various model outcomes responded differently to the variations of the parameters (Figure 4). The model had high sensitivities to the survival rate from feeding larvae to questing nymphs, systemic transmission efficiencies, the average number of larvae on one reservoir host, the average number of nymphs on one reproduction host, weekly mortality rate of questing nymphs, proportion of time-step spent in grassland for reproduction host and the density of reservoir hosts. The model was relatively insensitive to changes in the duration of interstadial development, survival rate from feeding nymphs to questing adults in woodland, weekly mortality rate of questing adults in woodland, and the movement capacity of reproduction host per week. The scaling factor of tick survival rates in grassland had an important impact in grassland only (not shown).

### Effects of Woodland Fragmentation on DON, NIP and DIN

Lyme disease risk indices differed greatly among scenarios (with varying woodland coverage and block size). By contrast, there were only small differences between the simulations for the five landscapes within each scenario (with fixed woodland coverage and block size but varying spatial arrangement; not shown).

**Figure 5:**
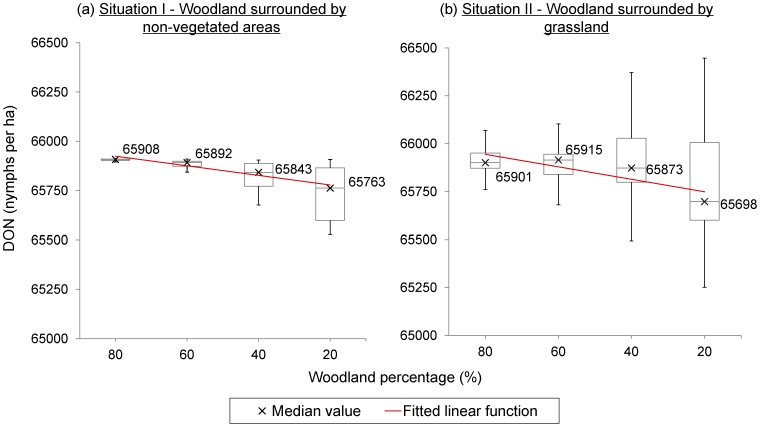
The effects of woodland coverage on densities of nymphs. Figure shows boxplots of densities of nymphs (DON) in woodland categorised by woodland percentages in situation I and II. The lower and upper boundaries of box refer to the 1^st^ and 3^rd^ quartiles of DON in each category. Crosses indicate the median value of DON. The whiskers refer to maximum and minimum DON values in each category. Red lines are two linear functions of woodland percentage categories on the median value of DON in situation I: (a) DON  =  −49 * woodland percentage categories +65973, R^2^  = 0.92; and in situation II: DON  =  −65 * woodland percentage categories +66009, R^2^  = 0.70.

**Figure 6:**
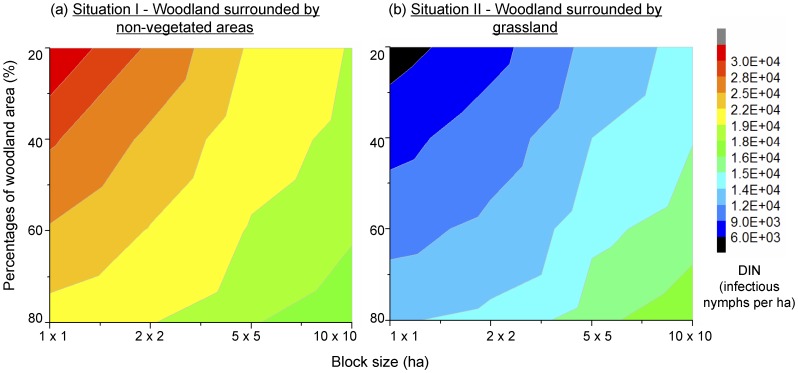
Density of infectious nymphs under different landscape fragmentation scenarios. Figure shows the simulated density of infectious nymphs (DIN) in woodland for landscapes with different woodland percentages and block sizes in the two situations.

The land cover adjacent to woodland had a major impact on risk indices observed in woodlands. In general, DON varied relatively little (65861±172 nymphs per ha in woodland), and hence DIN (17145±5888 infectious nymphs per ha in woodland) depended largely on NIP (26.04%±8.97% in woodland). DON in woodland increased as the woodland coverage increased regardless of surrounding land cover types (Figure 5). With increasing block size and woodland coverage, NIP and DIN decreased in woodland adjacent to non-vegetated area (situation I) but increased in woodland adjacent to grassland (situation II) (Figure 6). The contrast between the two situations was highest for landscapes with 20% woodland and 1×1 cell blocks with NIP and DIN were 85.20% and 85.18% lower in situation II. The contrast was lowest in landscapes with 80% of woodland cover arranged in 10×10 cell blocks with NIP and DIN dropping by 6.20% and 6.10%. In grassland, DON, NIP and DIN fluctuated. The peak values of NIP in grassland were higher than the NIP in woodland, however, DON and DIN in grassland were always lower than 0.1% of DON and DIN in woodland.

### Key Landscape Metrics to Lyme Disease Risks

**Figure 7:**
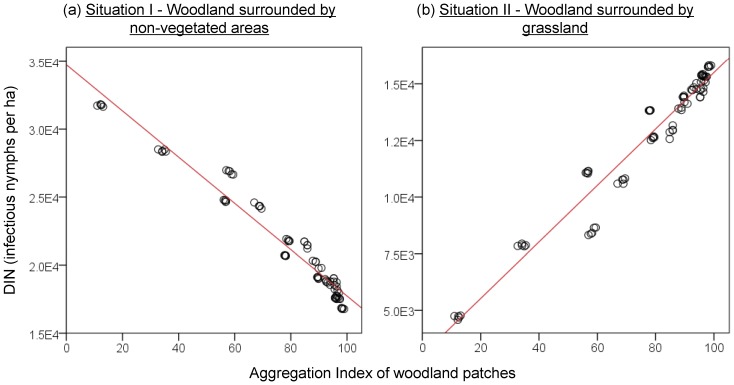
The effects of woodland aggregation index on densities of infectious nymphs. Red lines indicate two linear functions of aggregation index (AI) on the simulated densities of infectious nymphs (DIN) in woodland of: (i) DIN  =  −170 * AI +34745 in situation I, R^2^  = 0.96; and (ii) DIN  = 124 * AI+3031, R^2^  = 0.95 in situation II.

Landscape-level metrics of configuration are all significant predictors (P<0.05) of Lyme disease risk indices in woodland. In both situations, variations of NIP and DIN were well explained (*R^2^*>0.75 for all functions of involved landscape metrics in all fitting shapes). For robustness, an arcsine transformation was also tested [41], [42] for NIP. The regression results (not shown) were consistent with the linear results. The best models for NIP and DIN in woodland were linear functions of aggregation index that explained 95.6% and 94.8% of the DIN variance respectively in situation I and situation II (Figure 7). Patch density and landscape shape index were positively associated to DIN and NIP in situation I but negative in situation II. The aggregation index was negatively associated to risk indices in situation I but positively in situation II. The pattern of DON was explained at an adequate level (highest *R^2^* = 0.88) in situation I but at a limited level (highest *R^2^* = 0.13) in situation II. In both situations, patch density and shape index were negatively associated to DON while aggregation index was positively associated with it. In general, when woodlands were surrounded by non-vegetated areas, nymphal infections were higher in landscapes with longer and more irregular woodland patches and with lower aggregation level of woodland. However, when woodland was surrounded by grassland, the reverse applied.

## Discussion

Absolute values of the predicted densities depend strongly on the assumed relationship between dragging data and actual densities. Therefore, more emphasis should be put on the relative values than on the absolute values. Our model indicated a strong influence of woodland fragmentation on Lyme disease risk. Increasing fragmentation by decreasing coverage of woodland and decreasing size of blocks lead to (i) an increase of the NIP and DIN in woodlands adjacent to non-vegetated areas, and to (ii) a decrease of the NIP and DIN in woodlands adjacent to grasslands. The reason why grassland made such differences can be inferred from the parameters and transition rules utilised in the model. With lower tick survival and lower reproduction hosts availability, grasslands act as a sink for ticks. Lower survival of infectious ticks can contribute to lower infection prevalence in reservoir hosts in grassland. When questing larvae were picked up in grassland, the probability of getting infected would be lower. As a result, such larvae dropped off in woodland could dilute infection in woodland. Consistent with the negative association found by Guerra et al. [14] between grassland and *I. scapularis* ticks, our results emphasise the presence of grassland as a way of reducing the density and infection prevalence of *I. ricinus* tick in highly fragmented woodlands. It can therefore be hypothesised that strategies like burning or mowing the grassland, which can reduce tick abundance, may also amplify the Lyme disease risks in adjacent woodland. The important role of reproduction hosts on sustaining the local tick population can be highlighted as well. In situation II, DON was higher in landscapes with greater woodland covers, where reproduction host abundances at landscape level were higher. Similar positive relations were also found between questing *I. ricinus* abundances and deer densities in various habitat types [43], [44]. Our findings can provide important directions for future empirical studies, such as including the effects of adjacent land cover types, as previous analyses mostly focused on woodlands [15], [45]. In this study, we only examined fragmented woodland with two extreme situations of adjacent land covers: entirely grassland or entirely non-vegetated areas. In reality, woodland are often adjacent to mixed areas, consisting of non-vegetated areas and grassland, for example suburban residential (houses and gardens) and agricultural areas (pastures/croplands). Further insight could be gained by investing the effect of varying their relative proportion.

By further analysing the simulation results, we found significant impacts of landscape configuration metrics on Lyme disease risks. Based on these findings, two consequences of woodland fragmentation on host movement can be highlighted. First, movement of reproduction hosts between isolated forested patches favour pathogen transmission. Movements between forest patches were only possible when patches were closer than the movement capacity of the host. Thus, a higher patch density and a lower aggregation level can result in higher between-patch movement rates. In situation I, this led to higher nymphal infection prevalence in woodland. Theoretical studies have paid increasing attention to the effects of between-patch host movement on the invasion and persistence of disease [46], [47]. Population groups in small forest patches can be exposed to considerable risk of direct disease transmission if between-patch movement of reservoir host is sufficiently high. In line with Watts et al [48], our model expands such conclusion to the field of vector-borne diseases, suggesting reproduction host movement between isolated forest patches could also favour and maintain disease transmission.

Second, movements of hosts between different land cover types shape the local patterns of nymphal abundance and infection prevalence. In the sensitivity analysis, higher movements of reproduction hosts out of their habitat could lead to lower woodland DON. Simulation results by Gaff and Gross [49] and Hoch et al. [19] showed a similar decrease of tick density in forests when considering an increased deer movement to adjacent grassland. It can indicate the role of reproduction hosts in transporting ticks between land cover types. In situation II, movements of hosts between land cover types were related to the length of forest edge, which is a function of landscape shape index. Decreased movements of both reservoir and reproduction hosts to non-vegetated areas (associated to lower landscape shape index value) were related to an increase in woodland NIP. This would provide crucial hypotheses for future empirical studies: controlling the movements of reproduction host between different land cover types is unlikely to reduce the pathogen transmission. For instance, fencing the woodland may not be useful. Fencing has already been reported to be less useful to remove ticks in the forest or to control tick infestation in adjacent moorlands [44]. A possible reason can be that the local absence of a reproduction host may increase tick feeding on reservoir hosts [50]. This may lead to an increase of pathogen transmission.

Common processes related to changes in forest area or structure include forest harvesting and forest conversion. Our findings suggest possible impacts of forest management practices: if forest is exploited by removing (i) large clumps rather than a number of small blocks, (ii) regular shapes like square and round that minimize forest edge and (iii) those adjacent to areas that are already deforested, then the risk of Lyme disease may be lowered. Similar suggestions for forest harvesting have been proposed by [51]. Although this can lead to slower forest recovery, payoff may be found in the context of risk of tick-borne diseases.

Biological process models for tick-borne zoonoses are very few in numbers whilst the published empirical works are numerous. The situation indicates that a formal framework to understand and control the transmission of tick-borne zoonotic diseases has not been achieved. Models for tick population dynamics [17], [18], [19], [52] are quite detailed in tick biology but none have explicitly stated landscape heterogeneity. Models for pathogen dynamics [48], [49] adopted representations of landscape but assumed single-host processes or completely ignored the life stages of tick. This study, however, helps to bridge the gap. The present model for the dynamics of both tick and pathogen accommodates a multi-host structure and tick post-egg life stages onto a cell-based representation of the landscape. Cell-based representation of the landscape is strongly associated to remotely sensed data, which are abundant and provide detailed information on the landscape (e.g. spatial relationships, attributes etc.). Such data format may be more convenient to model complex and dynamic systems [53] than the patch-based approach.

In our model, several simplifying assumptions were made that may have influenced the results. These could be adapted in future developments to better represent reality. Firstly, the host preference of ticks could be more flexible. The present model simplifies tick feeding by assuming larvae only feed on reservoir host and adult tick only on reproduction hosts. Evidence that larvae also feed on reproduction hosts [54] and that adults feed on reservoir hosts [55] has been published recently. Moreover, in some instances, host-finding rates could be affected by density-dependent competition between ticks [56]. Secondly, movement patterns of host could be more detailed. The stochastic movement rules adopted in our model were only a first step. The trigger and completion of the movement may be multi-factorial, for example dependent on local host density, the phase of movement (i.e. home-ranging and dispersive) and geographical connectivity [57]. Thirdly, the influence of climate and seasonal weather patterns could be included. In tick-borne zoonoses transmission systems, the temporal dynamics of either pathogen or populations of tick and their hosts have been studied extensively [58], [59]. These temporal dynamics result from environmental factors such as seasonal changes of climate and host abundance [60], [61]. Some key tick behaviours have been regarded to be particularly sensitive to season, for example host-finding activity, diapause and maturation. Fourthly, biodiversity, which can be influenced by landscape fragmentation [62], may need to be considered. A very simple species assemblage of two generic hosts was adopted because the field data of species richness, distribution and abundance are largely missing in Belgium, and large uncertainties remain on reservoir capacity of various host species, as well as their tendency to pick up ticks. However, the changing diversity and composition of host community can in theory significantly affect the zoonotic dynamics [63]. When data for more species are available, more land cover types (for example, ecotones) need to be included as different species may prefer different types of the land cover [9] in which ticks also suffer from different mortality rates [22]. Finally, tick feeding behaviours could be better specified. In the model, we assumed fixed durations for tick feeding. However, it has been reported that the duration of feeding can vary, for example larvae sometimes feed for almost one week [23]. For computational reasons, a time step of one week was used, which affected the opportunities for larvae to spread. It may be important to explore the effect of this assumption, as well as whether or not the drop-off rhythms of ticks (i.e. the maximum drop-off of engorged ticks may occur during a specific period in a day) exists for *I. ricinus* and responses to temperature and land cover [64].

Cellular automata allow for a certain degree of flexibility in the theoretical study of complex ecosystems. For example in the tick-borne disease model at hand, applying a set of simplifying assumptions allowed to explore the effect of landscape structure. Beyond the immediate results brought by testing a range of scenarios, the model also allowed to outline a number of interesting areas of further empirical investigation. In this regard, the virtual setting of the model is an excellent complement to empirical work when field data are insufficient for direct comparisons [65], or, as is the case for ticks, extremely costly and challenging to collect. Still, further challenges will be encountered when applying the model to scenarios based on real-world situations. It is clear that, when transposing this model to real-world cases, more challenges may emerge, and require case-specific modifications.

In conclusion, the model developed in this study incorporated a cell-based representation of the environment to explore the effects of forest fragmentation on the risks of Lyme disease in a spatially-explicit manner. It can be combined with either real world landscapes or artificial representations. Assuming no impacts of biodiversity, our simulations have shown a strong influence of configuration of habitat patterns, i.e. the density, shape and aggregation level of woodland patches, on density and *B. burgdorferi* infection prevalence of *I. ricinus* ticks. These results suggest that, via altering the patterns of local host population interactions, landscape fragmentation can significantly affect the landscape-level dynamics of Lyme disease risk.

## Supporting Information

Appendix S1 Formulae for tick life cycle and disease transmission.
(DOC)

Appendix S2 Modelling host movement patterns in cellular automata.
(DOC)
