# Supplementary material for: Consequences of Landscape Fragmentation on Lyme Disease Risk: A Cellular Automata Approach
Source: PLoS One. 2012 Jun 25;7(6):e39612. doi: 10.1371/journal.pone.0039612 (PMC3382467; doi:10.1371/journal.pone.0039612)
Supplement: Appendix S1 — Formulae for tick life cycle and disease transmission. (DOC) [file pone.0039612.s001.doc]

**Appendix S1 Formulae for tick life cycle and disease transmission**

For each cell at time step *t*, we modelled:

1. Development of tick populations:

$qL_{t}=s^{qL}\cdot qL_{t-1}+\beta\cdot s^{AL}\cdot fA_{t-d^{AL}-1}-fL_{t-1}$ (1)

$qN_{t}=s^{qN}\cdot qN_{t-1}+s^{LN}\cdot fL_{t-d^{LN}-1}-fN_{t-1}^{H}-fN_{t-1}^{R}$ (2)

$qA_{t}=s^{qA}\cdot qA_{t-1}+s^{NA}\cdot(fN_{t-d^{NA}-1}^{H}+fN_{t-d^{NA}-1}^{R})-fA_{t-1}$ (3)

where *qL*, *qN* and *qA* indicate the questing populations of larvae, nymphs and adults. Superscripts *H* and *R* for nymphs (*N*) indicate the population is feeding on reproduction hosts and reservoir hosts respectively. *d^AL^, d^LN^* and *d^NA^* are development periods between feeding and moulting into the next life stages and *s^AL^, s^LN^* and *s^NA^* are tick survival rates in those periods. *s^qL^, s^qN^* and *s^qA^* are tick survival rates in feeding phases. All survival rates are 0.94 times lower in grassland than in woodland. *β* refers to the number of eggs per adult.

2. Disease transmission

a. Systemic transmission:

$\Delta fIL_{t}=\theta^{HT}\cdot\frac{IH_{t}}{H_{t}}\cdot(fL_{t}-fIL_{t})$ (4)

$\Delta fIN_{t}^{H}=\theta^{HT}\cdot\frac{IH_{t}}{H_{t}}\cdot(fN_{t}^{H}-fIN_{t}^{H})$ (5)

$\Delta IH_{t}=\theta^{TH}\cdot(fIL_{t}+fIN_{t}^{H})\cdot\frac{H_{t}-IH_{t}}{H_{t}}$ (6)

b. Trans-ovarial transmission:

$\Delta qIL_{t}=\beta\cdot s^{AL}\cdot fIA_{t-d^{AL}-1}\cdot\theta^{TE}$ (7)

where Δ*fIL*, Δ*fIN*, and Δ*IH* are the increased infectious populations of feeding larvae, feeding nymphs and reservoir hosts by systemic disease transmission. Δ*qIL* is the increased infectious population of questing larvae by trans-ovarial transmission. *θ^HT^* and *θ^TH^* indicate the efficiency of pathogen transmitting from reservoir hosts to ticks and from ticks to reservoir hosts. *θ^TE^* indicates the transmission efficiency from adults to their eggs.

c. Infectious populations of questing tick and reservoir host:

$qIL_{t}=s^{qL}\cdot(qIL_{t-1}+\Delta qIL_{t-1})-fIL_{t-1}$ (8)

$qIN_{t}=s^{qN}\cdot qIN_{t-1}+s^{LN}\cdot(\Delta fIL_{t-d^{LN}-1}+fIL_{t-d^{LN}-1})-fIN_{t-1}^{H}-fIN_{t-1}^{R}$ (9)

$qIA_{t}=s^{qA}\cdot qIA_{t-1}+s^{NA}\cdot(\Delta fIN_{t-d^{NA}-1}^{H}+fIN_{t-d^{NA}-1}^{H}+fIN_{t-d^{NA}-1}^{R})-fIA_{t-1}$ (10)

$IH_{t}=(1-r^{H})\cdot IH_{t-1}+\Delta IH_{t-1}$ (11)

where *qIL*, *qIN*, *qIA* and *IH* indicate infectious questing larvae, nymphs, adults and infectious reservoir hosts. *r^H^* is the removal rate of reservoir infections.
