# Supplementary material for: Consequences of Landscape Fragmentation on Lyme Disease Risk: A Cellular Automata Approach
Source: PLoS One. 2012 Jun 25;7(6):e39612. doi: 10.1371/journal.pone.0039612 (PMC3382467; doi:10.1371/journal.pone.0039612)
Supplement: Appendix S2 — Modelling host movement patterns in cellular automata. (DOC) [file pone.0039612.s002.doc]

**Appendix S2 Modelling host movement patterns in cellular automata**

Host movement can be modelled at different levels . In our study, we modelled the movement of host populations and focused on the distribution patterns.

In a cellular automata, we considered an extended Moore neighbourhood (Figure 1) for the home range of a host type (*X*). *X* can be a reservoir host (*H*) or a reproduction host (*R*). The vertical distance from the centre to the boundary of the neighbourhood denotes the host movement capacity *MC^X^*.


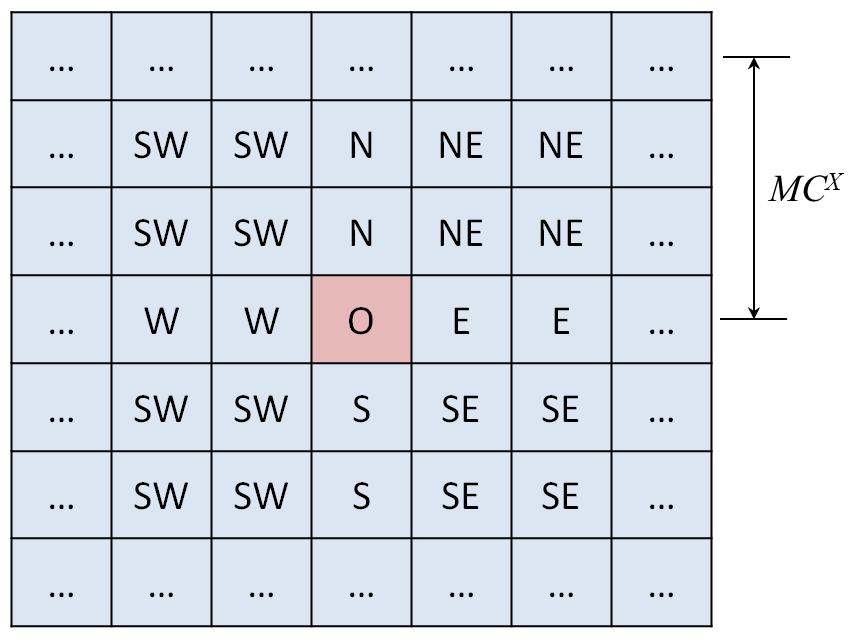


Figure 1 Extended Moore neighbourhood. Moore neighbourhood comprises the eight cells surrounding a central cell (*O*) on a square lattice, and the extended Moore neighbourhood reaches over the distance of the next adjacent cells. *MC^X^* refers to the host movement capacity. Thus the sizes of neighbourhood were different for reproduction hosts and reservoir hosts.

In an extended Moore neighbourhood, the eight directions are east, northeast, north, northwest, west, southwest, south, and southeast (Figure 1). We assumed that hosts have an equal probability to move in each of the directions. For each cell, the population of a host type (reservoir or reproduction) was evenly divided into eight parts that would be considered for movement in the eight directions. For each direction, we used (*i*,*j*) and (*x*,*y*) to respectively refer to the home cell and the destination cell. The second assumption was that host movements were always within their neighbourhoods (i.e. home ranges) . The following steps (I) and (II) were repeated eight times, once for each direction of the neighbourhood.

(I) *Trigger of movement*. In each direction, we considered two movement parameters *di* and *dj* composing the lengths of the projected movement on the two axis of the 2D space. For example, if the direction was “east”, then (*x*,*y*) = (*i+di*, *j+*0); if the direction was “northwest”, then (*x*,*y*) = (*i-di*, *j+dj*). *di* and *dj* were generated by taking absolute values of randomly generated numbers that followed a Gaussian distribution with mean of 0 and the standard deviation of *MC^X^*/3. Therefore, hosts had a higher probability to move to closer cells. The movement was triggered only if the length (i.e. the square root of *di^2^*+*dj^2^*) was larger than 100 m (size of one cell). As movements were assumed to be within hosts’ home ranges, values *di* and *dj* were adjusted to *MC^X^* if greater than *MC^X^*. Failed trigger of the movement in one direction means that the part of the population under consideration (1/8 of the total population in the cell) would remain in (*i*,*j*).

(II) *Completion of movement or return*. Once the movement in a direction was triggered, whether or not the movement could be completed depended on the land cover type of the destination cell (*x*,*y*):

1. If (*x*,*y*) was a habitat, then the movement in the direction succeeded. The 1/8 of the concerned hosts in (*i*,*j*) moved to (*x*,*y*). Accordingly, 1/8 of total and infectious feeding ticks in (*i*,*j*) were transported to (*x*,*y*). In the present model, this accounts for the reproduction hosts movement patterns in woodland and reservoir host movement patterns in both woodland and grassland.
2. If (*x*,*y*) was a non-habitat, then the movement in the direction failed and population would return.
   1. If (*x*,*y*) was a non-vegetated cell, then hosts returned directly and no ticks would be transported.
   2. If (*x*,*y*) was a grassland cell and the concerned host type was reproduction hosts, then the 1/8 of the concerned reproduction hosts remained in (*i*,*j*) at the end of the time step but assumed to have spent a proportion of the time step (*pG*) venturing in (*x*,*y*). Thus, *pG* of total and infectious feeding ticks on the 1/8 of the concerned reproduction hosts in (*i*,*j*) were transported to (*x*,*y*). Meanwhile, questing ticks in (*x*,*y*) may also feed on the venturing host population, thus *pG* of all potential feeding ticks in (*x*,*y*) were also transported to (*i*,*j*).

Reference

1. Tang W, Bennett DA (2010) Agent-based modeling of animal movement: A Review. Geography Compass 4: 682-700.

2. Fryxell JM, Hazell M, Borger L, Dalziel BD, Haydon DT, et al. (2008) Multiple movement modes by large herbivores at multiple spatiotemporal scales. P Natl Acad Sci USA 105: 19114-19119.
